# Comprehensive Characterization of Raw and Processed Quinoa from Conventional and Organic Farming by Label-Free Shotgun Proteomics

**DOI:** 10.1021/acs.jafc.4c08623

**Published:** 2025-01-17

**Authors:** Rocío Galindo-Luján, Laura Pont, Zoran Minic, Maxim V. Berezovski, Fredy Quispe, Victoria Sanz-Nebot, Fernando Benavente

**Affiliations:** † Department of Chemical Engineering and Analytical Chemistry, Institute for Research on Nutrition and Food Safety (INSA·UB), 16724 University of Barcelona, 08028 Barcelona, Spain; ‡ Serra Húnter Program, Generalitat de Catalunya, 08007 Barcelona, Spain; § John L. Holmes Mass Spectrometry Facility, Department of Chemistry and Biomolecular Sciences, 6363 University of Ottawa, Ottawa K1N 6N5, Ontario, Canada; ∥ National Institute of Agricultural Innovation (INIA), 15024 Lima, Peru

**Keywords:** conventional farming, nanoLC-MS/MS, organic farming, processed quinoa, raw quinoa, shotgun proteomics

## Abstract

Quinoa is widely recognized for its exceptional nutritional properties, particularly its complete protein content. This study, for the first time, investigates the effects of processing methods (boiling and extrusion) and farming conditions (conventional and organic) on the proteomic profile. Following a label-free shotgun proteomics approach, a total of 1796 proteins were identified and quantified across all quinoa samples. Regarding processing, both boiling and extrusion produced protein extracts with lower total protein content, with the number of identified proteins decreasing from 1695 in raw quinoa to 957 in processed quinoa. Boiling led to a reduction in protein diversity and expression, while extrusion, which involves high temperatures and pressures, specifically decreased the abundance of high molecular mass proteins. Concerning cultivation practices, organic farming was associated with a broader protein diversity, especially proteins related to translation (28 vs 5%), while conventional farming showed a higher abundance of catalytic and enzymatic proteins (67 vs 46%). These findings highlight the distinct proteomic changes induced by different processing methods and farming conditions, offering valuable insights to manage quinoa’s nutritional, bioactive, and functional properties across various production practices.

## Graphical abstract

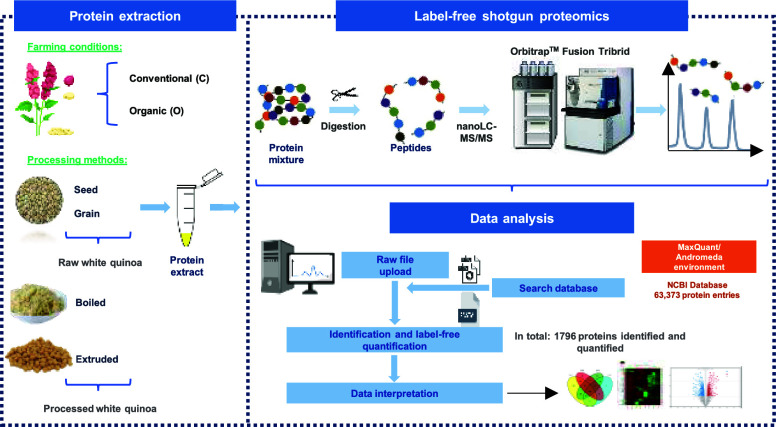

## 1 Introduction

Quinoa (Chenopodium quinoa Willd.) can be regarded as an excellent food choice due to its remarkable nutritional properties, particularly its high-quality protein composition with a well-balanced profile of essential amino acids.^1–3^ Additionally, unlike most cereals, quinoa is gluten-free and nonallergenic.^1,4^ In recent years, the outstanding nutritional benefits of quinoa, combined with its adaptability to diverse agroecological conditions, have led to a substantial increase in demand and the global expansion of its cultivation.^1,5,6^

Quinoa seeds^2,3^ need to be processed to improve digestibility and enhance their techno-functional properties, which are crucial for its industrial applications.^7^ After removing the pericarp and washing to eliminate saponins (responsible for the bitter taste), common processing methods such as boiling and extrusion are employed.^8–10^ Boiling consists of cooking the grains in an excess of water for around 15 min,^11^ while extrusion involves exposing quinoa grains to heat, mechanical energy, and pressure to shape the final product.^9^ Compared to alternatives like roasting or steam preconditioning, boiling and extrusion are simpler and more time-efficient. However, these heat and pressure treatments can alter quinoa’s physicochemical properties, often leading to protein denaturation, oxidation, and aggregation.^12,13^ The proteomic profile of quinoa can also be influenced by farming practices. With the growing consumer demand for more nutritious foods, organic farming has gained increased prominence. This practice, which avoids synthetic chemicals and promotes crop rotation,^14–16^ has contributed to the rise in organic quinoa consumption, particularly in regions such as the US, Australia, Canada, the EU, and traditional producers like Peru.^1,5,17^

The literature on quinoa proteomics is relatively recent and has mainly focused on raw seeds and grains. In our previous research, we described a shotgun proteomics approach to characterize four commercially available quinoa grains (black, red, and white quinoa from Peru, and white quinoa from Bolivia, also known as royal), allowing us to establish a comprehensive quinoa grain map comprising 1211 proteins.^18^ This study served as a groundwork for developing a simple data mining strategy aimed at identifying quinoa grain proteins with potential immunonutritional bioactivities, including those related to cancer.^19^ A recent study also described a shotgun proteomics approach to evaluate changes associated with water limitation (rainfed conditions) when compared to full irrigation (irrigated conditions) in quinoa seed samples, revealing a total of 2577 proteins.^20^ Other studies have demonstrated the usefulness of untargeted proteomics approaches for the characterization of quinoa proteins after subjecting nonedible parts of the plant, such as the leaves or guard cells, to mitovirus infection^21^ or salinity treatments.^22,23^ Nevertheless, none of the studies have explored the impact of different processing and farming procedures on the raw quinoa proteome.

In this study, we employed, for the first time, a label-free nano-liquid chromatography-tandem mass spectrometry (nanoLC-MS/MS) shotgun proteomics approach to extensively examine the proteome of both raw (seeds and grains) and processed (boiled and extruded) white quinoa (Salcedo variety) cultivated under conventional and organic farming conditions. The proposed methodology provides a comprehensive and detailed set of 1796 proteins, offering potential utility in enhancing the nutritional value of raw quinoa under diverse processing or farming conditions.

## 2 Materials and Methods

### 2.1 Chemicals

All of the chemicals were of analytical reagent grade or better. Sodium hydroxide (NaOH, ≥99.0%), hydrochloric acid (HCl, 37% (v/v)), boric acid (H_3_BO_3_, ≥99.5%), β-mercaptoethanol (≥99.0%), sodium dihydrogen phosphate (NaH_2_PO_4_, ≥99.0%), water (LC-MS grade), acetonitrile (ACN, LC-MS grade), bovine serum albumin (BSA, relative molecular mas (*M*_r_) of 66,000), formic acid (FA, 99.0%), 4-(2-hydroxyethyl)-1-piperazineethanesulfonic acid (HEPES, ≥99.5%), urea (≥99.0%), Triton X-100 (laboratory grade), glycerol (≥99.5%), tris­(2-carboxyethyl)­phosphine hydrochloride (TCEP, ≥98.0%), sodium dodecyl sulfate (SDS, ≥99.8%), and iodoacetamide (IAA, ≥99.0%) were provided by Merck (Darmstadt, Germany). Trypsin/Lys-C enzyme mix (MS grade) was supplied by Promega (Madison, WI).

### 2.2 Sample Treatment

White quinoa seeds (Salcedo variety, National Institute of Agricultural Innovation of Peru) were cultivated in 2018 under conventional and organic conditions in La Molina, Lima, Peru (latitude 12° 04′ 36″S, longitude 76° 56′ 43″W, altitude 241 m above sea level (masl)) and in Omas, Lima, Peru (latitude 12° 33′ 25.6″S, longitude 76° 19′ 9″W, altitude 1227 masl), respectively. In conventional soil fertilization, a mixture of urea, diammonium phosphate, and potassium chloride was applied. In contrast, organic soil fertilization employed “bokashi”, a fermented food-based fertilizer prepared with ingredients such as animal dung, molasses, and other organic materials. To separate the grain from the pericarp, 3 kg of quinoa seeds were polished for 5 min using a scarifier machine (Vulcano, Lima, Peru). After that, the obtained quinoa grains (2.8 kg) were washed 3 times for 5 min in a quinoa-to-water ratio of 1:10 (m/v) at room temperature (rt). Finally, the washed quinoa grains were dried at 40 °C in an oven (Memmert, Schwabach, Germany) and stored at rt in a dry environment.

#### 2.2.1 Boiling Process

250 g of white quinoa grains from both conventional and organic farming were ground with an ultracentrifugal mill (Restch, Schwabach, Germany) at 18,000 rpm for 30 s. The sieving operation was conducted by using a mesh with a 0.5 mm opening during the grinding process. 50 g of the resulting flour was dispersed in water before boiling to prevent lump formation, ensuring a homogeneous mixture. A flour-to-water mixture (1:20, m/v) was heated in a cooking pot for 20 min at 100 °C with continuous stirring. After the process, the boiled flour was cooled for 20 min, dried at 40 °C for 72 h, and subsequently stored in polyethylene (PE) bags at rt until further analysis.

#### 2.2.2 Extrusion Process

1.5 kg of white quinoa grains from both conventional and organic farming were extruded using a corotating twin screw extruder (Inbramaq, São Paulo, Brazil). The extruder comprises a feeding zone, a heating zone, and a die zone. The overall length of the extruder barrel was 960 mm, with a screw diameter of 30 mm and a cylindrical die diameter of 10 mm. The temperature was configured as follows: the extruder feeding zone was set at 30 °C, progressing to 40 °C and, then, 50 °C. The heating zone exhibited variations at 70, 85, and 100 °C, while the die zone was maintained at temperatures of 100, 110, and 125 °C. The grain feeding rate was set at 14 kg/h, with the screw speed held constant at 800 rpm. The retention time was maintained between 10 and 15 s, and the cut frequency was configured at 17 Hz. After the process, the extruded grains were cooled for 15 min and subsequently stored in PE bags at rt until further analysis.

#### 2.2.3 Protein Extraction

Proteins from raw (i.e., seeds and grains), boiled, and extruded quinoa from conventional (C_seed_, *C*_grain_, C_boiled_, and C_extruded_) and organic farming (O_seed_, O_grain_, O_boiled_, and O_extruded_) were extracted as in our previous work,^24^ with some modifications (two independent replicates were extracted for each quinoa sample). Briefly, 250 mg of each sample was mixed with 2 mL of water and 39 μL of 1 M NaOH (final pH of 10.0) using a vortex Genius 3 (Ika, Staufen, Germany) for 3 h at rt. Separation of soluble proteins from the insoluble residue was performed by centrifugation at 23,000*g* for 60 min at 4 °C in a cooled Rotanta 460 centrifuge (Hettich Zentrifugen, Tuttlingen, Germany). For protein purification, the supernatant pH was adjusted with 22 μL of 1 M HCl to obtain a final pH value of 5.0. After centrifugation at 30,000*g* for 30 min at 4 °C, precipitated proteins were resuspended in 1 mL of a solution of 60 mM H_3_BO_3_ (pH adjusted to 9.0 with NaOH). The resulting solution was filtered through 0.22 μm nylon filters (MSI, Westboro, MA) before analysis. All pH measurements were made using a Crison 2002 potentiometer and a Crison electrode 52–03 (Crison Instruments, Barcelona, Spain).

### 2.3 Total Protein Content Analysis

The total amount of protein in the quinoa extracts was estimated spectrophotometrically using a capillary electrophoresis (CE) instrument equipped with a diode-array detector (7100 CE, Agilent Technologies, Waldbronn, Germany). Two independent replicates from C_seed_, C_grain_, C_boiled_, C_extruded_, O_seed_, O_grain_, O_boiled_, and O_extruded_ quinoa were injected at 50 mbar for 10 s using a 58 cm total length (*L*_T_) × 50 μm internal diameter (i.d.) × 365 μm outer diameter (o.d.) fused silica capillary (Polymicro Technologies, Phoenix, AZ). A calibration curve was established by analyzing BSA standard solutions at concentrations ranging between 100 and 1000 μg/mL. Flow injection experiments were carried out without voltage, mobilizing the sample plug by applying 50 mbar of pressure after the injection. Absorbance was measured at 214 nm from the area of the detected protein peaks.

### 2.4 Proteolytic Digestion

Quinoa protein extracts were digested using a modified filter-aided sample preparation (FASP) protocol designed for proteomic analysis.^25^ In this process, 50 μg of protein sample was diluted to a volume of 100 μL using a denaturation buffer consisting of 8 M urea and 25 mM HEPES (pH 8.0). After vortexing briefly, samples were transferred to 10,000 *M*_r_ cutoff (MWCO) centrifugal filters (Millipore, Molsheim, France). The sample volume was reduced to 20 μL through centrifugation for 20 min at 14,000*g*, followed by protein reduction with the addition of 4 mM TCEP in 100 μL of denaturation buffer. Incubation at 25 °C for 30 min was followed by a 15 min centrifugation step at 14,000*g*. Proteins were then alkylated using 20 mM IAA in 100 μL of denaturation buffer, followed by a 40 min incubation at 25 °C and a 15 min centrifugation at 14,000*g*. Subsequently, 100 μL of digestion buffer (0.6% (v/v) glycerol and 25 mM HEPES, pH 8.0) were added to the filter and, after a 15 min centrifugation at 14,000*g*, the filter was transferred to a clean collection tube. Proteolytic digestion was achieved by adding MS-grade trypsin/Lys-C mix at an enzyme-to-protein ratio of 1:300 (m/m), followed by incubation in the dark under shaking at 600 rpm at 37 °C for 12 h. Peptides were separated in the filtrate by centrifugation at 14,000*g* for 15 min, and digestion was stopped by adding 1% (v/v) FA and centrifuged for 2 min at 15,000*g*. The digested proteins collected from the supernatant were desalted using disposable TopTip C-18 columns (Glygen, Columbia, MD), evaporated to dryness, and reconstituted in 20 μL of water containing 1% (v/v) FA.

### 2.5 NanoLC-MS/MS

NanoLC-MS/MS analyses were performed on an Ultimate3000 nanoRLSC instrument (Thermo Scientific) coupled to an Orbitrap Fusion Tribrid instrument (Thermo Scientific). 2 μL of protein digests were injected and separated on a column (15 cm L_T_ × 75 μm i.d. × 365 μm o.d. fused silica capillary, Polymicro Technologies) packed in-house with Luna C18 particles (Luna C18(2), 3 μm, 100 Å, Phenomenex, Torrance, California). Mobile phase solvents were (A) water with 0.1% (v/v) FA and (B) ACN with 0.1% (v/v) FA, working at a flow rate of 0.30 μL/min (0–7 min, 2–2% B; 7–107 min, 2–38% B; 107–112 min, 38–98% B; 112–122 min, 98–98% B; 122–130 min, 98–2% B; 130–140 min, 2–2% B). The mass spectrometer was operated in electrospray ionization (ESI) positive mode under the following parameters: ion source temperature of 250 °C, ion spray voltage of 2.1 kV, and Top Speed mode enabled for rapid alternation between full scans and MS/MS scans. Full-scan MS spectra were acquired with a resolution of 60,000 over 350–2000 *m*/*z*. Precursor ions were selectively filtered through monoisotopic precursor selection, considering a charge state range of +2 to +7, and dynamic exclusion parameters (30 s with a ± 10 ppm window). The automatic gain control was configured to 5 × 10^5^ for full scans, while an intensity threshold of 5 × 10^3^ was applied for MS/MS scans. Fragmentation was achieved using collision-induced dissociation (CID) in a linear ion trap. Isolation of precursors utilized a 2 *m*/*z* isolation window, followed by fragmentation with a normalized collision energy set at 35%.

### 2.6 Data Analysis

MaxQuant (Thermo Scientific, version v1.6.17.0)^26^ in combination with the search engine Andromeda^27^ was used for protein and peptide identification in all of the MS/MS raw files (detailed parameters for automated qualitative analysis are provided in Table S1A). Trypsin was selected as the proteolytic enzyme, permitting a maximum of two missed cleavages, peptide charges spanning from +2 to +7, a 10 ppm precursor mass tolerance, and a 0.5 Da fragment mass tolerance. In addition, search parameters were set to allow for dynamic modifications, including methionine oxidation and acetylation on the N-terminus. The search database consisted of a nonredundant quinoa protein sequence FASTA file containing the 63,370 entries from Chenopodium quinoa found in the reference sequence (RefSeq) project from The National Center for Biotechnology Information database (NCBI, https://www.ncbi.nlm.nih.gov/). Normalized label-free quantification (LFQ) values were obtained by applying the in-built MaxLFQ algorithm (detailed parameters for label-free quantitative analysis are provided in Table S1B).^28^

Data interpretation was conducted through the use of Venn diagrams, distribution bar graphs, heat maps, volcano plots, and gene ontology (GO) classification graphs. Specifically, Venn diagrams were generated considering the number of identified proteins using the VennDiagram R package (version 1.7.3) (for further details, refer to the tutorial at https://www.datanovia.com/en/blog). Distribution bar graphs were constructed considering the percentage of identified proteins within different *M*_r_ ranges (below 20,000, between 20,000 and 40,000, between 40,000 and 60,000, between 60,000 and 80,000, between 80,000 and 100,000, and above 100,000). The construction of the heat maps was achieved considering the LFQ values of the identified proteins through the freely available web server Heatmapper (http://www.heatmapper.ca). Volcano plots were generated considering the LFQ values of the identified proteins through the use of different freely available R packages, including tidyverse (version 2.0.0) for data manipulation and visualization, ggpubr (version 0.6.0) for plot generation, and rstatix (version 0.7.2) for *t* test statistical analyses (https://www.datanovia.com/en/blog). These plots displayed the log_2_ fold-change (FC: calculated as the ratio of mean LFQ values between two conditions, represented as condition 1–condition 2) on the *x*-axis and the -log 10 *p*-values on the *y*-axis, with proteins meeting the criteria of FC > 1.5 and *p*-values <0.05 deemed significant. GO analyses were performed using the PANTHER classification system (http://www.pantherdb.org). However, as C. quinoa is not available in PANTHER, which works primarily with UniProt identifiers and modeled organisms, the NCBI accession numbers (IDs) of the identified proteins were blasted against the UniProt database (https://www.uniprot.org/) of Arabidopsis thaliana, a model plant organism (average percent identity was 72 ± 16%).

## 3 Results

### 3.1 Optimization of the Protein Extraction Method

In our previous work,^24^ we employed a simple protein extraction method, which consisted of solubilizing proteins at pH 10.0, followed by a 1 h incubation at 36 °C, isoelectric precipitation at pH 5.0, and subsequent redissolution of the protein precipitate in 60 mM H_3_BO_3_ at pH 9.0. Unfortunately, minimal protein was extracted from boiled and extruded quinoa samples due to protein denaturation from heat and pressure treatments.^11,29^ To address this, an alternative extraction solvent described in the literature was explored,^8,29,30^ which consisted of a water solution containing 0.035 M NaH_2_PO_4_ (pH 7.0), 0.1 M 2-mercaptoethanol, and 1.5% (v/v) SDS. However, protein extractability was not improved. Finally, the best results were obtained by making some adjustments to our previously described method, i.e., increasing the water volume in the extraction solvent, extending the incubation time to 3 h, and augmenting speed rates and time during the centrifugation steps. Under the optimized protocol, the total protein content analysis yielded the following values (*n* = 2): 5.5 ± 0.1% (m/m) for C_seed_, 4.58 ± 0.05% (m/m) for C_grain_, 0.9 ± 0.1% (m/m) for C_boiled_, 1.1 ± 0.1% (m/m) for C_extruded_, 5.3 ± 0.2% (m/m) for O_seed_, 4.5 ± 0.1% (m/m) for O_grain_, 0.47 ± 0.01% (m/m) for O_boiled_, and 0.56 ± 0.03% (m/m) for O_extruded_.

### 3.2 Label-Free Shotgun Proteomics Analyses

In this study, we used an Orbitrap Fusion Tribrid mass spectrometer, as in our previous work with commercially available grains.^18^ To optimize sample preparation and separation, we implemented a FASP protocol and extended the chromatographic gradient. Protein identification and label-free quantification were performed using the MaxQuant/Andromeda environment, combined with a nonredundant quinoa protein sequence FASTA file containing 63,370 entries from the RefSeq NCBI database. A total of 1796 quinoa proteins were successfully identified across all quinoa samples (including 169 uncharacterized proteins), which represents an improvement over our previous study with commercial quinoa grains, where 1211 proteins were identified.^18^ Table S2 provides detailed information about the protein group level, the ID, the protein name, *M*_r_, the Andromeda score, the number of peptides, the sequence coverage, and the normalized LFQ intensity for the 1796 quinoa proteins identified in the studied samples. It is worth mentioning that for every quinoa sample only proteins found in the two replicates were reported. Furthermore, the analysis complied with stringent quality standards, with a peptide-spectrum math false discovery rate (PSM FDR) and protein FDR exceeding 99%, ensuring high confidence in peptide and protein identifications (Table S1A). As can be observed in Table S2, the 1796 quinoa proteins were identified at the group level with different reliabilities, with Andromeda score values ranging between 323 and 2.

### 3.3 Comprehensive Interpretation of the Proteomics Data

#### 3.3.1 Venn Diagrams

For a simple representation of the results, two Venn diagrams were created. Figure 1A illustrates the relationship between the number of identified proteins in raw quinoa (seeds and grains) cultivated under both conventional and organic farming. Notably, a greater number of proteins were identified in organic raw quinoa (1637 proteins considering both O_seed_ and O_grain_) compared to conventional raw quinoa (1320 proteins considering both C_seed_ and C_grain_). Likewise, the number of identified proteins was only slightly greater for the seeds compared to that of the grains. Among these proteins, 945 were identified across all of the samples, while 750 were present in only some of them. Regarding proteins identified in only one sample, 31 proteins were exclusively identified in C_seed_, 11 in C_grain_, 186 in O_seed_, and 60 in O_grain_. Moving to Figure 1B, which depicts the relationship between the number of identified proteins in processed quinoa (boiled and extruded) cultivated under both conventional and organic farming, a notable reduction in the number of identified proteins compared to raw quinoa was observed (a total of 957 vs 1695 proteins, Figure 1B,A, respectively). Furthermore, as can be seen in Figure 1B, a greater number of proteins were identified in extruded quinoa (898 proteins considering both C_extruded_ and O_extruded_) compared to that in boiled quinoa (388 proteins considering both C_boiled_ and O_boiled_). In contrast, almost no differences were observed in the number of identified proteins considering organic and conventional farming. Among these proteins, 176 were identified in all of the samples, while 781 were only present in some of them. Regarding proteins identified in only one sample, 28 proteins were exclusively identified in C_boiled_, 85 were exclusively identified in C_extruded_, 13 were identified in O_boiled_, and 92 were identified in O_extruded_. All of these observations suggested differences at the proteome level between conventional and organic raw quinoa seeds and grains, especially after boiling and extruding quinoa grains.

**Figure 1:**
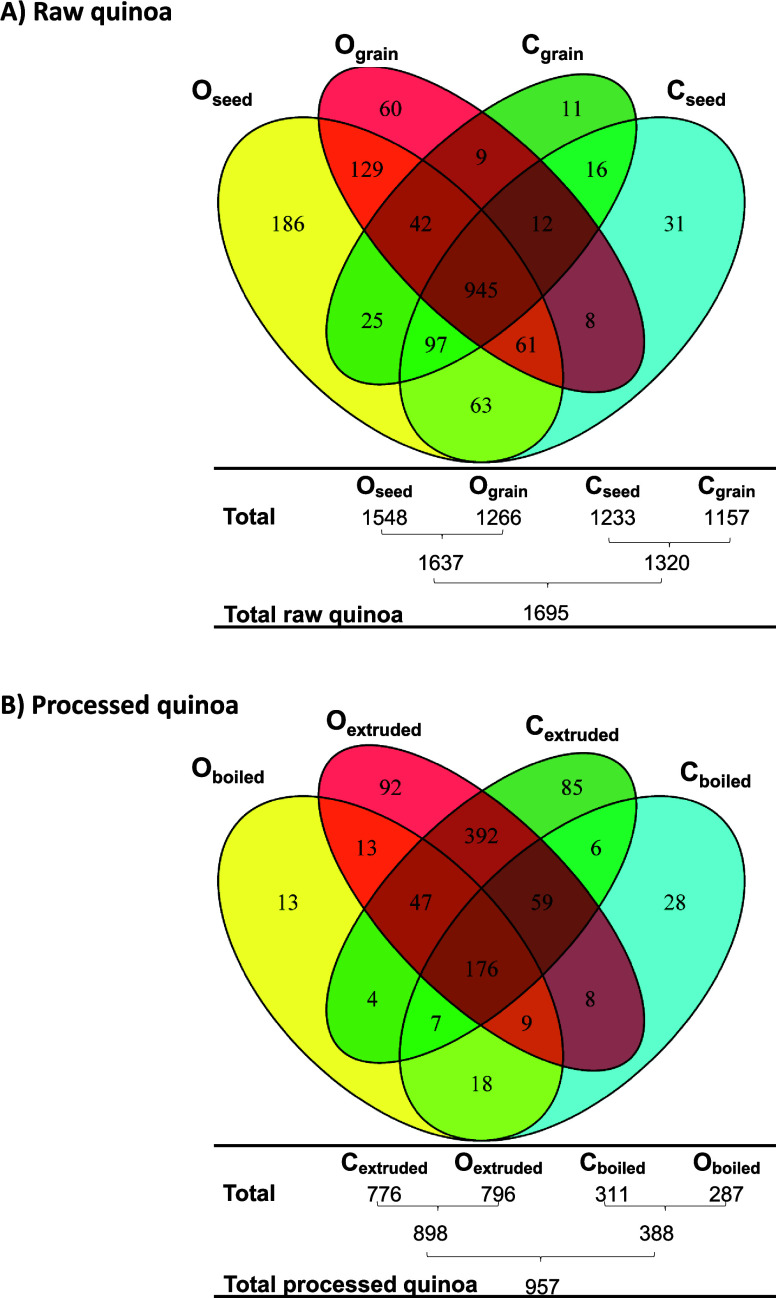
Venn diagram analysis of the identified proteins in (A) raw quinoa (including C_seed_, C_grain_, O_seed_, O_grain_) and (B) processed quinoa (including C_boiled_, C_extruded_, O_boiled_, O_extruded_). C: conventional farming, O: organic farming.

#### 3.3.2 Distribution Bar Graphs

In order to assess differences in the *M*_r_ protein profile between the studied quinoa samples, a distribution bar graph was constructed considering the percentage of identified proteins in all of the sample classes at different *M*_r_ ranges (Figure 2). As can be seen in Figure 2, raw and boiled quinoa samples from both conventional and organic farming (C_seed_, C_grain_, C_boiled_, O_seed_, O_grain_, and O_boiled_) predominantly exhibited proteins with *M*_r_ ranging between 20,000 and 40,000 (34, 34, 33, 35, 36, and 33%, respectively) and between 40,000 and 60,000 (28, 27, 25, 27, 27, and 24%, respectively). It is important to note that these two *M*_r_ ranges encompass the major storage quinoa seed proteins, including 11S globulins (*M*_r_ around 36,000), 7S globulins (*M*_r_ around 46,000), and 13S globulins (*M*_r_ around 55,000) (Table S2).^18,20^ Regarding extruded quinoa samples from both conventional and organic farming (C_extruded_ and O_extruded_), they predominantly exhibited proteins with *M*_r_ between 20,000 and 40,000 (37 and 37%, respectively), and below 20,000 (34 and 30%, respectively), highlighting a notable disparity in the *M*_r_ protein profile when subjecting quinoa grains to extrusion processes. This emphasizes the idea that extrusion processes, which are subjected to higher temperatures and pressures than boiling procedures, are more prone to induce protein unfolding and denaturation of higher *M*_r_ proteins (>40,000), hence poorer solubilities, recoveries, or bioavailabilities.^11,31^

**Figure 2:**
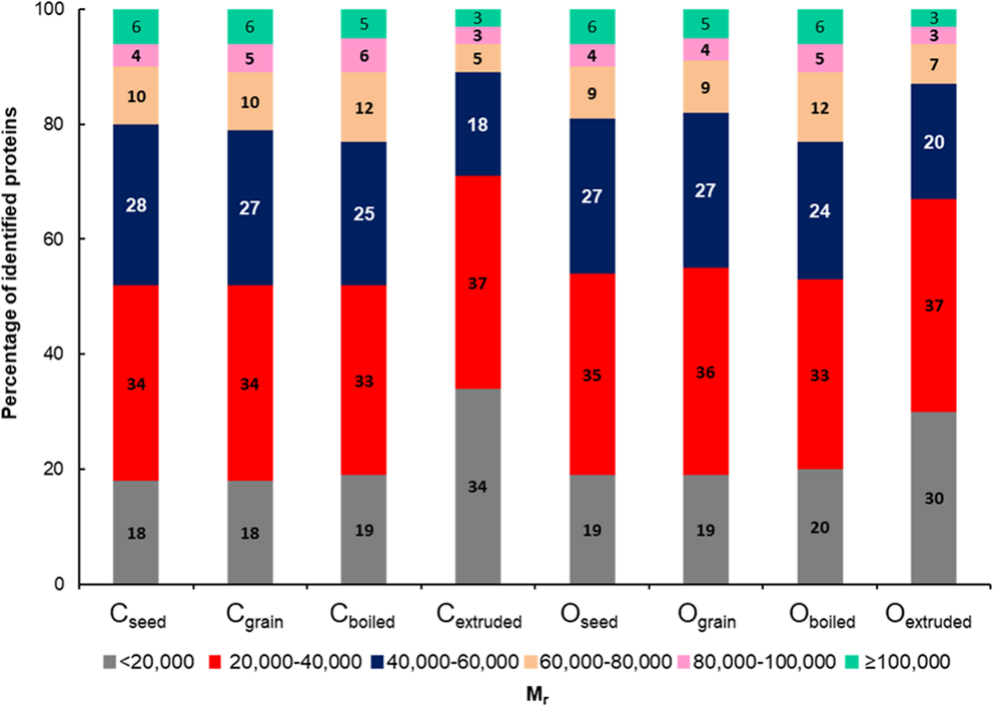
Distribution bar graph constructed considering the percentage of identified proteins in the studied quinoa samples within different *M*_r_ ranges (below 20,000, between 20,000 and 40,000, between 40,000 and 60,000, between 60,000 and 80,000, between 80,000 and 100,000, and above 100,000). C: conventional farming, O: organic farming.

#### 3.3.3 Heat Maps

To achieve a more precise distinction between the samples, differences in protein concentrations were considered. A heat map was created using the data matrix of average normalized LFQ intensities for the 174 proteins (represented in rows) identified across all quinoa samples (columns).^32–34^ As can be observed in Figure 3, each sample exhibited a distinctive protein concentration profile, with green, red, and black boxes representing upregulated, downregulated, and unchanged expression proteins, respectively. The dendrograms depicted in the heat map revealed that according to their protein concentration profile, raw (seeds and grains) and processed (boiled and extruded) quinoa samples were separated into two differentiated groups, regardless of the farming conditions. Within raw quinoa, C_grain_ and C_seed_ quinoa samples were clustered together, followed by O_seed_ and, finally, O_grain_ quinoa, which, according to the clusters, was the least closely related sample based on the quantified protein groups. Within processed quinoa, C_boiled_-O_boiled_ and C_extruded_-O_extruded_ were clustered together, suggesting a notable change in the protein concentration profile between boiled and extruded quinoa samples, regardless of the farming conditions. This observation supported our previous findings with the Venn diagrams and the distribution bar graph, where boiled and extruded samples presented a small percentage of common proteins and a different *M*_r_ protein profile.

**Figure 3:**
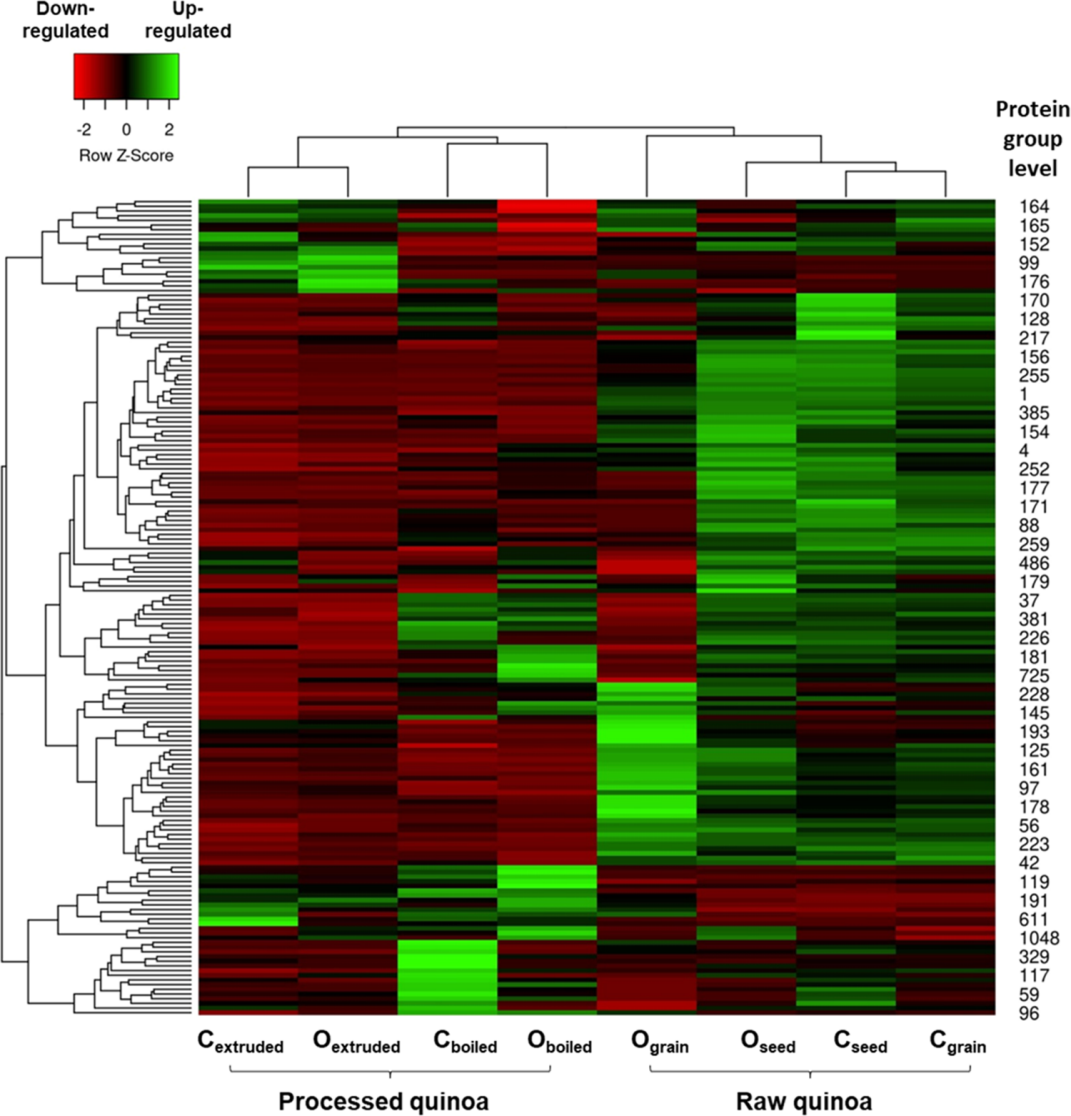
Heat map obtained using the row z-score normalized LFQ intensities of the identified proteins in the studied quinoa samples (C_seed_, C_grain_, O_seed_, O_grain_, C_boiled_, C_extruded_, O_boiled_, O_extruded_). C: conventional farming, O: organic farming.

#### 3.3.4 Volcano Plots

The application of the previously described data interpretation tools revealed that the most significant differences between conventional and organic farming practices were evident in raw quinoa samples without any significant distinctions between seeds and grains. This observation prompted the creation of a dedicated volcano plot for the C_raw_ – O_raw_ comparison (Figure 4A). To further explore variations associated with the processing methods (i.e., boiling and extrusion), additional volcano plots were generated based on the differentiated clusters observed in the heat map (Figure 3): Raw-Boiled (including samples from both conventional and organic farming, Figure 4B) and Raw-Extruded (including samples from both conventional and organic farming, Figure 4C). Table S3 shows the protein group level, the ID, the protein name, and the protein expression (upregulated in condition 2 (“+”), upregulated in condition 1 (“–”), and nonstatistically significant (“n.s.”)) for the quinoa proteins represented in the different volcano plots (C_raw_ – O_raw_, Raw-Boiled, and Raw-Extruded, Figure 4A–C). Examining Figure 4A, which distinguishes between conventional and organic farming in raw quinoa samples, it was determined that, out of the total 1262 represented proteins, 109 were upregulated in C_raw_ (green dots, “–” symbol in Table S3), 72 were upregulated in O_raw_ (red dots, “+” symbol in Table S3), and 1081 were considered nonstatistically significant for the differentiation (gray dots, “n.s.” acronym in Table S3). In the Raw-Boiled comparison (Figure 4B), out of the total 381 represented proteins, 166 displayed overexpression in raw quinoa, while a considerably lower number (22) exhibited overexpression in boiled quinoa (Table S3). A similar pattern was noted in Raw-Extruded (Figure 4C), where out of the total 822 represented proteins, 284 demonstrated upregulation in raw quinoa, whereas a lower number (152) were upregulated in extruded quinoa (Table S3). The results obtained from the volcano plots revealed quantitative variations in protein abundance between raw quinoa samples cultivated under conventional and organic farming, showing a comparable number of different proteins upregulated in both conditions. In addition, there were quantitative variations in protein abundance between raw and processed quinoa samples, notably indicating the downregulation of protein expression in processed quinoa, especially after boiling.

**Figure 4:**
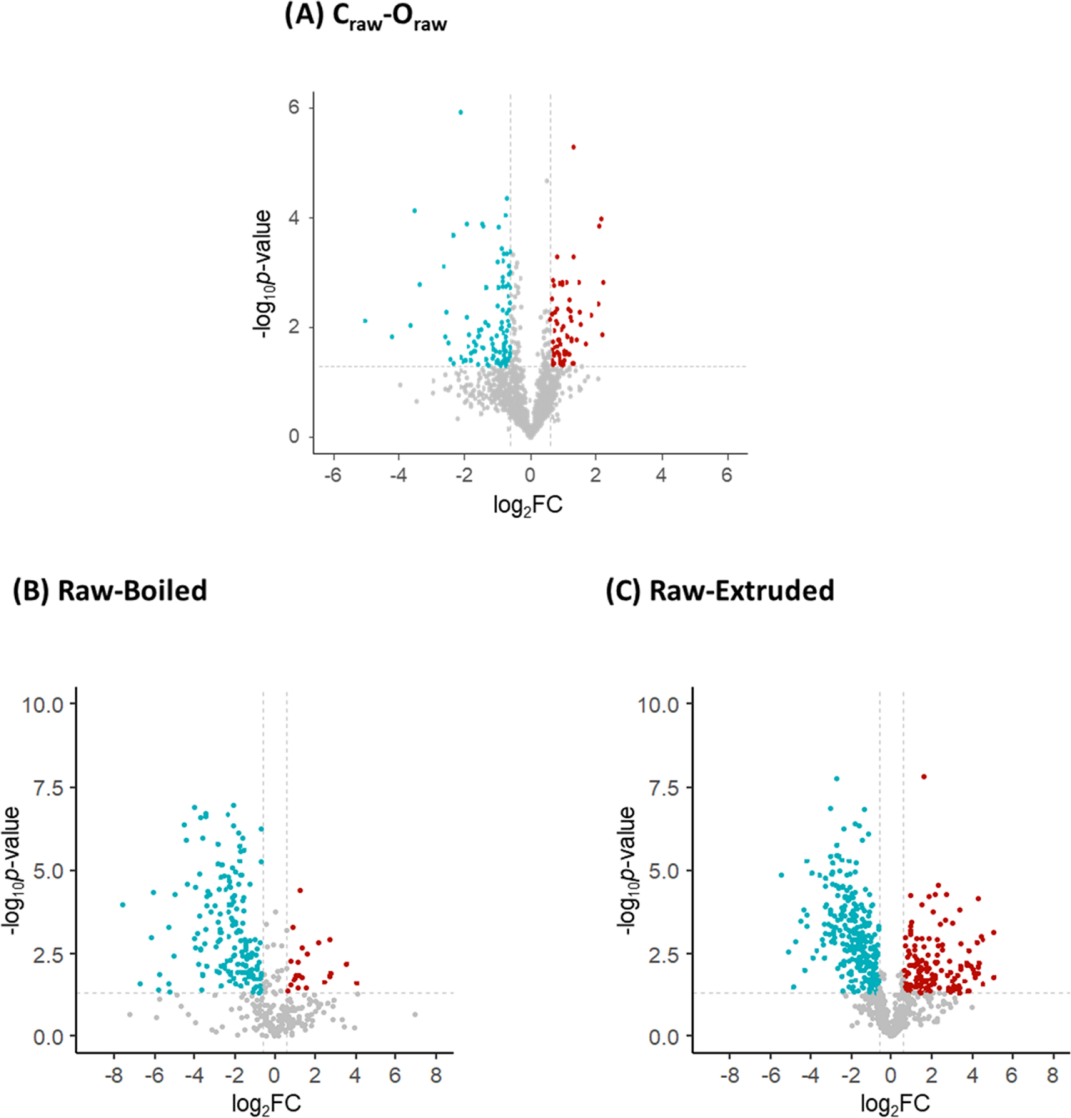
Volcano plots for discriminating between two conditions, represented as condition 1–condition 2: (A) C_raw_ – O_raw_, (B) Raw-Boiled (including samples from both conventional and organic farming), and (C) Raw-Extruded (including samples from both conventional and organic farming). *X*-axes represent the log_2_fold-change (log_2_FC) values (FC calculated as the ratio between the average LFQ values for the two compared conditions), and *Y*-axes depict the -log *p*-values (computed using statistical R packages). Only proteins with FC > 1.5 and ρ-values <0.05 are considered statistically significant for the differentiation. Upregulated proteins in condition 1 are represented as green dots (“–” in Table S3), upregulated proteins in condition 2 are represented as red dots (“+” in Table S3), and nonstatistically significant proteins are represented as gray dots (“n.s.” in Table S3). C: conventional farming, O: organic farming. Raw quinoa includes seeds and grains.

#### 3.3.5 GO Classification Graphs

Based on the results from the volcano plots, we conducted GO analysis at the molecular function (Figure S1), biological process (Figure S2), and protein class level (Figure S3) for the following groups: upregulated proteins in C_raw_ and O_raw_ (A), upregulated proteins in raw and boiled quinoa (B), and upregulated proteins in raw and extruded quinoa (C). It is important to note that only UniProt IDs corresponding to proteins upregulated under the conditions highlighted in the volcano plots were included in the GO analysis (these proteins are listed in Table S4).

In the molecular function category (Figure S1), comparing C_raw_ – O_raw_ (Figure S1A), the highest number of hits in both conditions were associated with catalytic and binding activities. However, the percentage of proteins with catalytic activities was significantly higher in C_raw_, while that of O_raw_ showed a broader range of less represented activities. Comparing raw-boiled (Figure S1B), great differences emerged. While raw quinoa exhibited a higher number of hits associated with catalytic and binding activities, boiled quinoa only showcased hits linked to binding, transcription regulator, and structural molecule activities. Comparing Raw-Extruded (Figure S1C), the greater number of hits in raw quinoa were associated with catalytic activities, closely followed by binding activities. In contrast, extruded quinoa exhibited the opposite trend. Concerning the less represented hits and in comparison to boiled and extruded quinoa, raw quinoa showcased proteins with a wider variety of molecular functions. These observations suggested that quinoa grain processing and, especially, boiling greatly deplete enzymes involved in catalytic activities and decrease protein variety.

In the biological process category (Figure S2), minimal differences were noted in all of the comparisons. Analyzing C_raw_ – O_raw_ (Figure S2A), the majority of hits in both classes were predominantly associated with metabolic and cellular processes. This pattern persisted in Raw-Boiled (Figure S2B) and Raw-Extruded (Figure S2C). In this case, no clear-cut trends affecting biological processes were identified, likely due to the highly heterogeneous protein classes involved in these biological processes.

Finally, the protein class category (Figure S3) showed notable differences. In the C_raw_ – O_raw_ comparison (Figure S3A), most hits in C_raw_ were classified as metabolite interconversion enzymes and protein-modifying enzymes, whereas in O_raw_, they were primarily classified as metabolite interconversion enzymes and translational proteins. This suggested that organic farming was associated with a broader protein diversity, especially proteins related to translation (translational proteins in Figure S3A, 28 vs 5%), while conventional farming showed a higher abundance of catalytic and enzymatic proteins (metabolite interconversion enzymes and protein-modifying enzymes in Figure S3A, 67 vs 46%). The prevalence of enzymatic protein classes in C_raw_ supports our earlier finding of enhanced catalytic activity, which may have nutritional, bioactivity, and techno-functional implications.^35^ In the Raw-Boiled (Figure S3B) and Raw-Extruded (Figure S3C) comparisons, a trend similar to the molecular function category emerged, with a decrease in protein diversity and enzymatic functions after processing, especially boiling.

## 4 Discussion

Processing methods, including boiling and extrusion, significantly impacted quinoa’s proteome, as evidenced by a reduction in the total protein content and the number of identified proteins in processed samples compared to raw quinoa. Extrusion, involving higher temperatures and pressures, caused notable shifts in the molecular protein profile, with a marked decrease in high M_r_ proteins (>40,000) and an increase in smaller proteins (<20,000), supporting the hypothesis that extrusion promotes protein denaturation and unfolding, potentially reducing solubility and bioavailability. Although less severe, boiling led to the depletion of catalytic and enzymatic proteins and a decrease in the diversity of molecular functions, as confirmed by the GO analysis. These processing effects were further reflected in the quantitative differences in protein expression highlighted by volcano plot analysis, where downregulation of proteins was observed, particularly after boiling. Raw quinoa retained a higher number of proteins involved in enzymatic and metabolic activities, which are likely more sensitive to thermal denaturation. Notably, both boiled and extruded quinoa exhibited distinct protein expression profiles, as indicated by heat map clustering, underscoring the unique effects of each processing method. The influence of farming practices on quinoa protein profiles was highlighted by Venn diagram analysis, which revealed a greater number of identified proteins in quinoa cultivated under organic farming compared to conventional farming, particularly in raw quinoa. Organic farming appeared to favor a broader protein diversity, as indicated by the GO analysis, which linked proteins upregulated in organic quinoa to a wider range of molecular functions, including translational activities. This suggests that organic farming may trigger different stress responses or metabolic pathways. In contrast, conventional farming demonstrated a higher representation of catalytic and enzymatic activities, which could have implications for the overall quality and properties of these proteins.

The observed changes in protein diversity and abundance may have significant implications for the quinoa’s nutritional, bioactive, and techno-functional properties. The depletion of storage proteins (11S, 7S, and 13S globulins) and enzymatic proteins, including amylases, proteases, lipases, hexokinases, ATP synthases, and polymerases (see Table S2), may compromise protein quality and digestibility, as these proteins are vital for providing essential amino acids and supporting digestion.^36^ Processing methods such as boiling and extrusion can denature or degrade these proteins, diminishing their solubility and digestibility, which in turn can reduce nutrient bioavailability and lower quinoa’s nutritional and bioactive value. In contrast, the proteomic differences observed between quinoa cultivated under organic and conventional farming systems shed light on the influence of agroecological conditions. Organic farming, characterized by natural soil fertility and reduced synthetic inputs, appears to impose abiotic and biotic stressors on the plant. These stressors may activate specific stress-response pathways, resulting in the upregulation of translation-related proteins, such as ribosomal proteins, initiation and elongation factors, and aminoacyl-tRNA synthetases, among others (see Table S2). These proteins are essential for the synthesis of new proteins in cells, potentially enhancing protein quality, bioactivity, and the production of bioactive compounds beneficial for human health.^37^ Conversely, conventional farming, with its reliance on chemical fertilizers and pesticides, may favor metabolic efficiency, as reflected in the higher abundance of catalytic and enzymatic proteins.

In conclusion, by combining insights from different data visualization tools (e.g., Venn diagrams, heat maps, volcano plots, and GO analyses), this study provides a multifaceted perspective on the impact of processing methods and farming conditions on the quinoa proteome. The integration of these approaches allows for a detailed understanding of protein composition and expression, paving the way for optimizing quinoa cultivation and processing to enhance its functional and nutritional value.

## Supplementary Material





## Data Availability

The mass spectrometry proteomics data have been deposited to the ProteomeXchange Consortium via the PRIDE partner repository with the data set identifier PXD050043.

## Notes

GO graphs classified by molecular function for the quinoa proteins upregulated in C_raw_ – O_raw_, Raw-Boiled, and Raw-Extruded (Figure S1); GO graphs classified by biological process for the quinoa proteins upregulated in C_raw_ – O_raw_, Raw-Boiled, and Raw-Extruded (Figure S2); and GO graphs classified by protein class for the quinoa proteins upregulated in C_raw_ – O_raw_, Raw-Boiled, and Raw-Extruded (Figure S3) (PDF)
List of parameters used for MaxQuant automated qualitative analysis and label-free quantitative analysis (Table S1); protein group level, ID, protein name, *M*_r_, Andromeda score, number of peptides, sequence coverage, and normalized LFQ intensity for the 1796 identified proteins in the studied quinoa samples after applying label-free shotgun proteomics (Table S2); protein group level, ID, protein name, and protein expression (upregulated in condition 2 (“+”), upregulated in condition 1 (“–”), and nonstatistically significant (“n.s.”)) for the quinoa proteins represented in the different volcano plots (Table S3); and protein group level, ID, and protein name for the 1627 quinoa proteins (derived from the total of 1796 identified proteins after excluding the 169 uncharacterized proteins detailed Table S1) and its correlation with 1527 Uniprot IDs from A. thaliana (with the corresponding percent identity) (Table S4) (XLSX)
