# Supplementary material for: Comprehensive Characterization of Raw and Processed Quinoa from Conventional and Organic Farming by Label-Free Shotgun Proteomics
Source: J Agric Food Chem. 2025 Jan 17;73(4):2669–77. doi: 10.1021/acs.jafc.4c08623 (PMC12164343; doi:10.1021/acs.jafc.4c08623)
Supplement: Supplementary file 1 [file jf4c08623_si_001.pdf]

Supporting information

Comprehensive Characterization of Raw and Processed Quinoa from Conventional and Organic Farming by Label-Free Shotgun Proteomics

Rocío Galindo-Luján, [†] Laura Pont, [*,†,‡] Zoran Minic, [§] Maxim V. Berezovski, [§] Fredy Quispe, [‖] Victoria Sanz-Nebot [†] and Fernando Benavente [†]

[†]Department of Chemical Engineering and Analytical Chemistry, Institute for Research on Nutrition and Food Safety (INSA·UB), University of Barcelona, 08028 Barcelona, Spain

[‡]Serra Húnter Program, Generalitat de Catalunya, 08007 Barcelona, Spain

[§]John L. Holmes Mass Spectrometry Facility, Department of Chemistry and Biomolecular Sciences, University of Ottawa, Ottawa K1N 6N5, Ontario, Canada

[‖]National Institute of Agricultural Innovation (INIA), 15024 Lima, Peru

[*]Email: laura.pont@ub.edu. Tel: (+34) 934039123. Fax: (+34) 93402123.

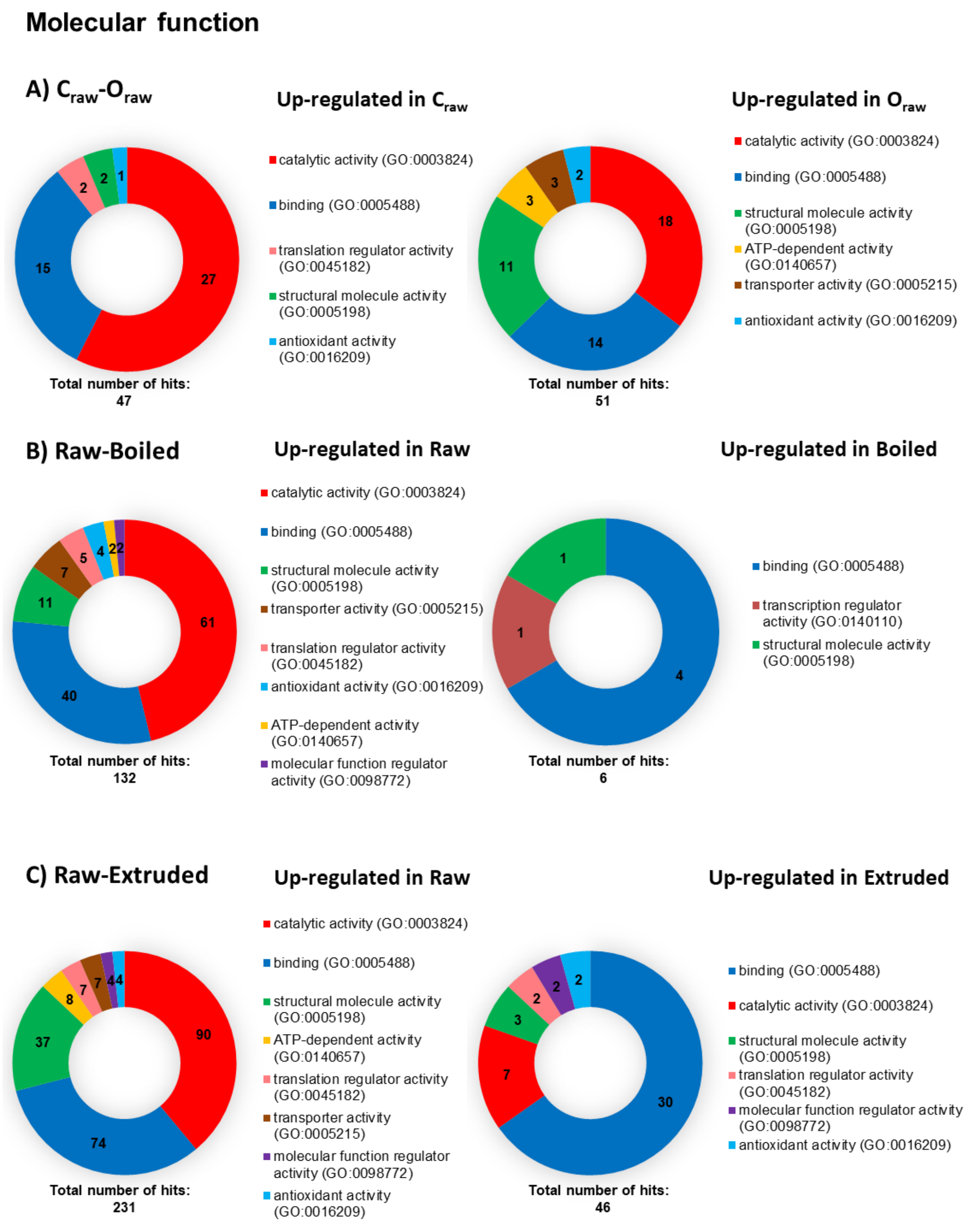

Figure S1. Gene Ontology (GO) graphs classified by molecular function for the quinoa proteins up-regulated in (A) C_{raw}-O_{raw}, (B) Raw-Boiled, and (C) Raw-Extruded, and blasted against the Uniprot database of *Arabidopsis thaliana*. C: conventional farming, O: organic farming. Raw quinoa includes seeds and grains.

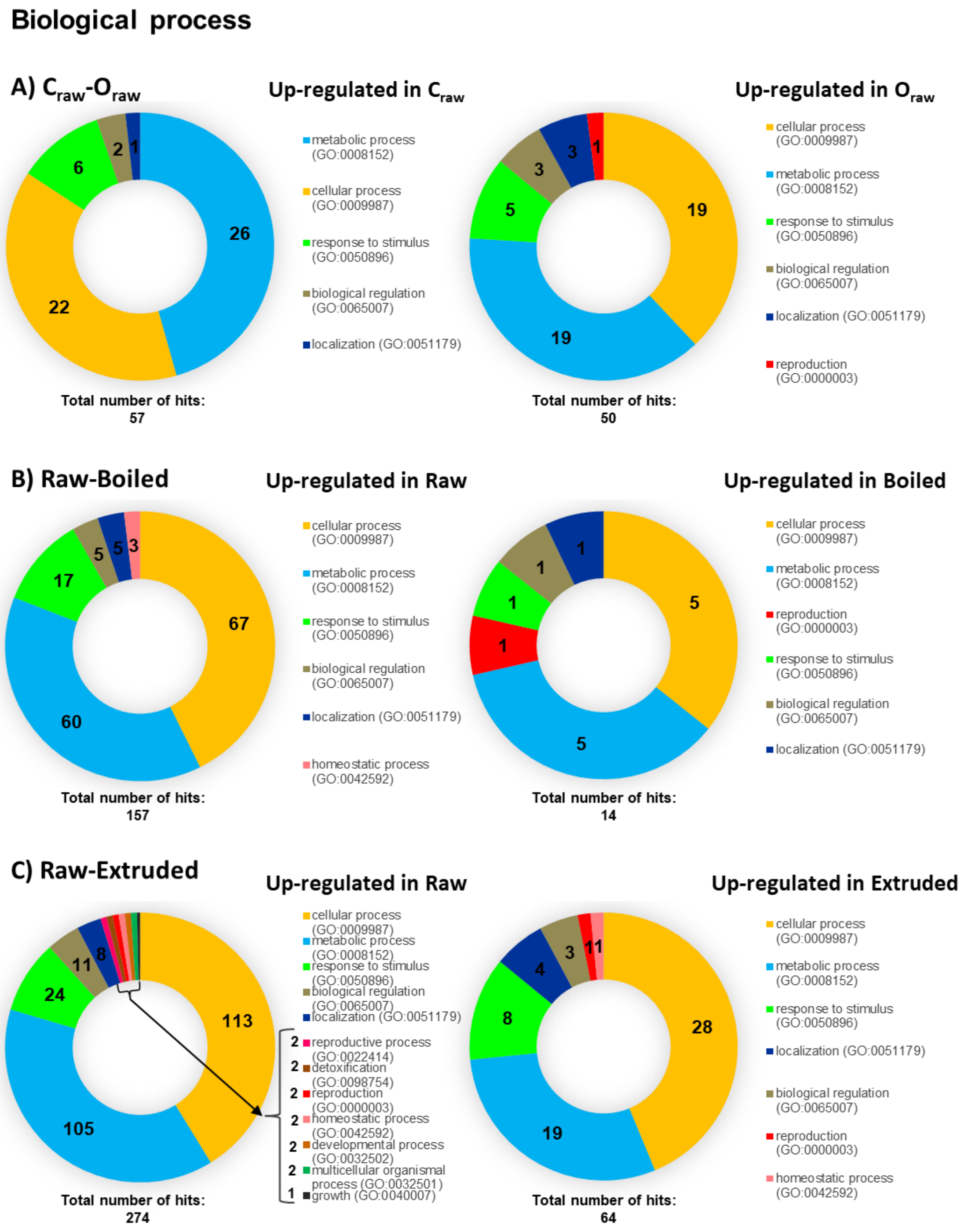

Figure S2. Gene Ontology (GO) graphs classified by biological process for the quinoa proteins up-regulated in (A) C_{raw}-O_{raw}, (B) Raw-Boiled, and (C) Raw-Extruded, and blasted against the Uniprot database of *Arabidopsis thaliana*. C: conventional farming, O: organic farming. Raw quinoa includes seeds and grains**.**

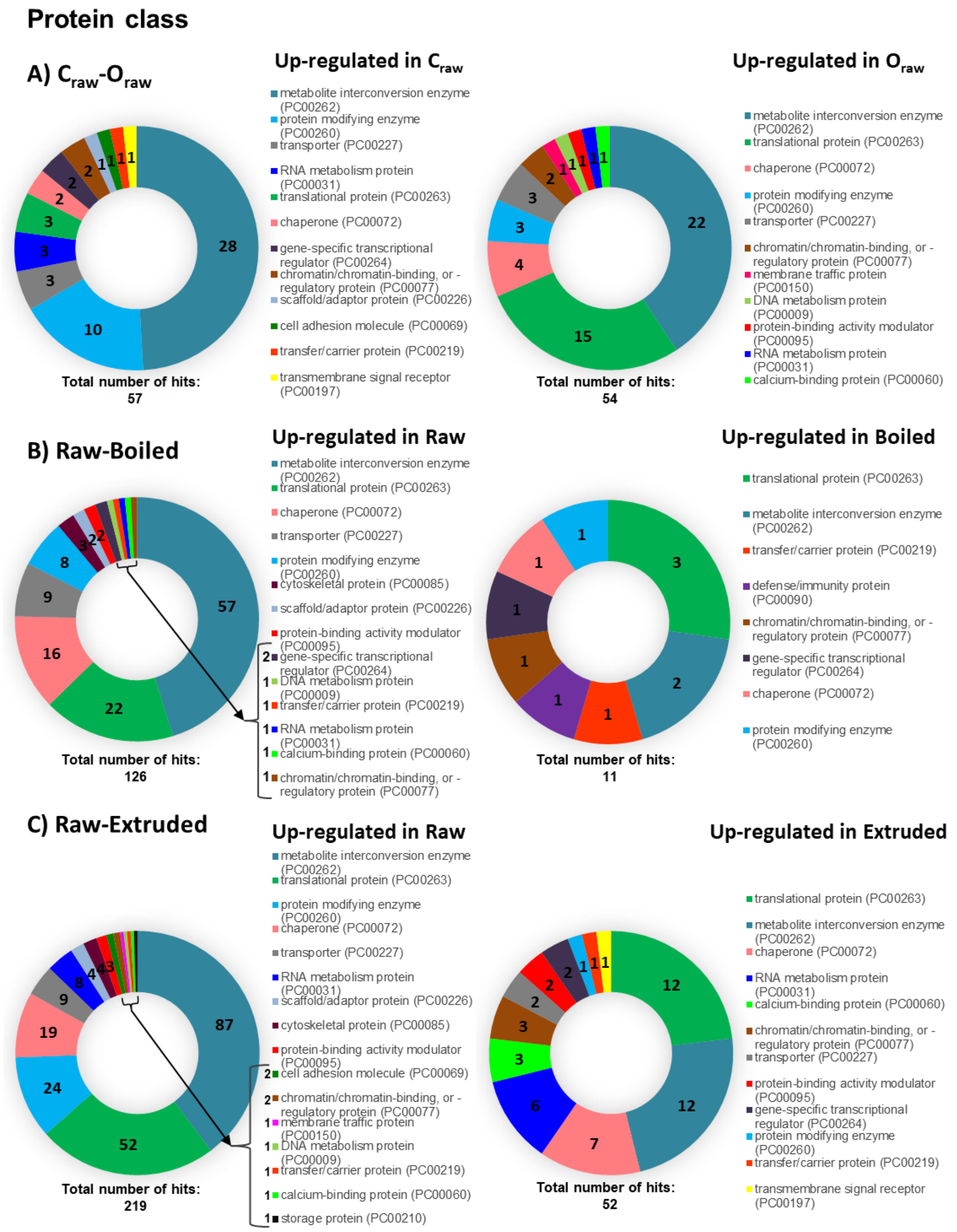

Figure S3. Gene Ontology (GO) graphs classified by protein class for the quinoa proteins up-regulated in (A) C_{raw}-O_{raw}, (B) Raw-Boiled, and (C) Raw-Extruded, and blasted against the Uniprot database of *Arabidopsis thaliana*. C: conventional farming, O: organic farming. Raw quinoa includes seeds and grains.
